# Comparison of Microsatellite Instability With Clinicopathologic Data in Patients With Colon Adenocarcinoma

**DOI:** 10.7759/cureus.57814

**Published:** 2024-04-08

**Authors:** Emine Cesmecioglu Karavin, Zeynep Sağnak Yılmaz, Hilmi Yazici, Safak Ersoz, Sevdegul Mungan

**Affiliations:** 1 Pathology, Marmara University Pendik Training and Research Hospital, Istanbul, TUR; 2 Pathology, Karadeniz Technical University Faculty of Medicine, Trabzon, TUR; 3 General Surgery, Marmara University Pendik Training and Research Hospital, Istanbul, TUR

**Keywords:** msh2, msh6, pms2, mlh1, mmr proteins, microsatellite instability, colorectal cancer

## Abstract

Background

Microsatellite instability (MSI) is a genetic condition caused by errors in DNA repair genes that cause colorectal cancer (CRC). The literature contradicts the frequency of MSI in sporadic CRCs and its effect on prognosis. This study investigated the distribution of clinicopathologic features and the relationship between MSI and survival outcomes.

Methodology

This is a retrospective study of 101 consecutive cases of CRC and immunohistochemical studies. All cases were retrospectively reviewed and reevaluated by histological grade, lymphovascular invasion, perineural invasion, tumor borders, dirty necrosis, tumor-infiltrating lymphocytes (TILs), Crohn’s-like lymphoid reaction, mucinous and medullary differentiation, and tumoral budding from pathological slides. An immunohistochemical study was performed in appropriate blocks for using MLH-1, MSH-2, MSH-6, and PMS-2. We collected the clinical stage, pathological tumor stage, lymph node metastasis, age, sex, tumor diameter, distant metastasis, localization, and survival information from patients’ clinical data.

Results

There was no statistically significant difference between the two groups regarding age, gender, tumor diameter, histological grade, tumor border, dirty necrosis, TILs, N and M stage, perineural and lymphovascular invasion, mucinous differentiation, medullary differentiation, and tumor budding characteristics of the patients. The MSI-H group was more frequently located in the right colon and transverse colon (p < 0.001), and the T stage was higher among them than in the MSI-L group (p = 0.014). Upon multivariate regression analysis, MSI status had no significant effect on survival time. Age and stage N and M were independent prognostic factors for colon cancer prognosis.

Conclusions

Our study presented the distribution of clinicopathological features and their relationship with MSI for 101 regional CRC patients. MSI status was detected by immunohistochemistry. Identifying MSI in CRCs may help personalize therapy planning. As the distribution of the features may vary from population to population, further investigations are needed on this topic.

## Introduction

Colorectal cancer (CRC) is considered one of the most prevalent malignancies in developed countries. It is the third most prevalent cancer across the world and the third leading cause of death, following lung and breast cancer [1].

Approximately 12%-15% of CRCs develop via the microsatellite instability (MSI) pathway [2]. This pathway involves the production of DNA mismatch repair genes, including *MLH-1*, *PMS-2*, *MSH-6*, and *MSH-2*. Identification of CRCs that develop via MSI has been considered clinically important [2,3]. This is mainly because patients with CRC involving MSI are associated with a better prognosis. These patients are more responsive to chemotherapy using 5-fluorouracil [4].

MSI identification is based on molecular analysis. The MSI status of a tumor may be classified as stable (MSS), low instability (MSI-L), or high instability (MSI-H). In hereditary non-polyposis CRC syndrome, MSI-H is caused by an inherited mutation in one of the mismatch repair genes (often *MLH-1* or *MSH-2*). For sporadic MSI-H CRCs, the primary mechanism for developing the mutator phenotype is the inactivation of the *MLH-1* gene by promoter hypermethylation [5]. Molecular analysis is considered a time-consuming, expensive method and requires specialized equipment. Therefore, routine testing in local laboratories is complex and requires referral to select centers. Immunohistochemical analysis of *MLH-1*, *PMS-2*, *MSH-2*, and *MSH-6* is a faster and easier method, and it is used as a routine test in laboratories.

Specific clinical and histopathologic features are expected to be monitored in cases of MSI-associated CRC. These include female sex, right colon localization, the tendency for multiple foci, lymphocytic reaction, mucinous and medullary differentiation, and high-grade morphology [6-8]. Therefore, there has been a recent emphasis on using clinicopathologic and immunohistochemical features to select patients for genetic analysis [9-11].

In this study, we aimed to investigate the clinical and pathological differences in the features among the two groups and the effect of MSI on CRC prognosis.

## Materials and methods

We retrospectively enrolled 116 patients who underwent colon resection between 2014 and 2016 in Karadeniz Technical University Faculty of Medicine (KTUFM), Department of Pathology, Trabzon, Turkey. The necessary permission was obtained from the Ethics Council of KTUFM (approval number: 2015/183). Patient demographic characteristics, including age and sex, tumor type, localization, size, lymphovascular invasion status, degree of differentiation, and tumor T, N, and M status of patients with adenocarcinoma, were recorded. At least three slides with hematoxylin and eosin (H&E) staining from each tumor were re-examined under a Nikon Eclipse E200 microscope to evaluate tumor borders, presence of dirty necrosis, tumoral budding, tumor-infiltrating lymphocytes (TILs), and Crohn’s-like lymphocytic reaction (CLR). Small blue mononuclear cells with a typical halo around them were considered TILs on the slides with H&E staining. Only the cells among the tumor cells were counted, whereas the cells between tumor stroma were not included in the count. Apoptotic cells were excluded from the count. The tumor area with the highest TIL density was counted at 10 consecutive high-magnification fields (×40) on a Nikon Eclipse E200 microscope. The cutoff value was set to 3. TILs were considered none (0), mild to moderate (1-2), and significant (3 and more). CLR and tumor budding were detected in the specimen, representing the tumor’s most invasive area. The largest lymphoid aggregate diameter ≥1 mm was considered positive for CLR. Any number of clusters consisting of a maximum of five cells separated from the tumor within the desmoplastic stroma were accepted as evidence of tumor budding. MSH2, MLH1, MSH6, and PMS2 antibodies were performed using a Ventana BenchMark ULTRA automated stainer (Ventana Medical Systems, Inc.). The intact diffuse and focal staining of all four antibodies was considered preservation of expression and regarded as MSI-L. The loss of expression of at least one of the immunostains was considered MSI-H.

Statistical analysis

Mean, standard deviation, median, minimum, maximum, interquartile range, frequency, and ratio values were used to express descriptive statistics of the data. An independent-sample t-test and a Mann-Whitney U test were used to analyze independent quantitative data. The chi-square test was used to analyze qualitative independent data. Univariate and multivariate Cox regression and Kaplan-Meier methods were used for survival analysis. SPSS version 28.0 software (IBM Corp., Armonk, NY, USA) was used in the analysis. A p-value ≤0.05 was considered statistically significant.

## Results

A total of 116 patients with colon adenocarcinoma diagnosed based on resection material between 2014 and 2016 in our laboratory were analyzed. Upon immunohistochemical evaluation of CRC cases in our study, no significant results were obtained with MLH-1 in 14 cases (negative internal control), while PMS-2 could not be examined in the same patients. In one case, where other markers were examined, no significant result was obtained with MLH-1. The remaining 101 cases were included in the study.

**Figure 1:**
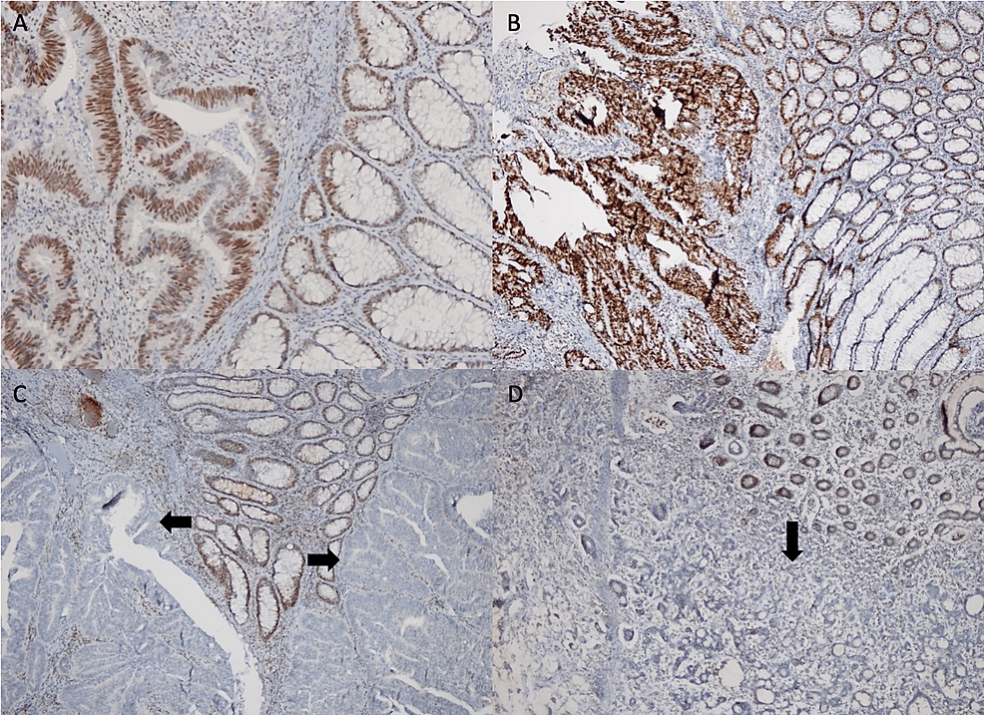
A: MLH-1 expression preserved in the tumor area and adjacent mucosal transition. B: PMS-2 expression preserved in the tumor area and adjacent mucosal transition. C: Tumor with loss of MLH-1 nuclear expression (arrow) and positive internal control. D: Tumor showing loss of PMS-2 nuclear expression (arrow) and internal positive control. (magnification ×100).

Of the 101 cases in which the quad panel could be examined, 72 had preserved expression of four markers. In 29 cases, expression was lost in at least one immune marker (Figure 1). The patients were examined in two groups, namely, MSI-L and MSI-H.

There was no significant difference between the two groups regarding age, gender, tumor diameter, histological grade, tumor border, dirty necrosis, TILs, mucinous differentiation, medullary differentiation, and tumor budding (Table 1). However, the incidence of CIL reaction was significantly higher in the MSI-L group (p = 0.041).

**Table 1:** Basic characteristics of the groups. *: SD: standard deviation; **: IQR: interquartile range. MSI: microsatellite instability

Clinicopathological features	MSI-Low 72 (71%), n (%)	MSI-High 29 (29%), n(%)	P-value
Age (years)(mean ± SD*)	61.9 (±13.2)	60.5 (±11.6)	0.288
Gender (%)			0.159
Male	44 (61%)	22 (76%)	
Female	28 (39%)	7 (24%)	
Tumor diameter (cm) (median (IQR**))	4 (3)	4 (2)	0.24
Histological grade			0.33
Grade 1	62 (86%)	24 (83%)	
Grade 2	7 (10%)	5 (17%)	
Grade 3	3 (4%)	0 (0%)	
Border			0.633
Infiltrative	60 (83%)	23 (79%)	
Expansive	12 (17%)	6 (21%)	
Dirty necrosis	57 (79%)	23 (79%)	0.987
Tumor-infiltrating lymphocytes			0.904
None	39 (54%)	17 (59%)	
Mild to moderate	23 (32%)	8 (28%)	
Significant	10 (14%)	4 (13%)	
Crohn’s-like lymphocytic reaction	14 (19%)	1 (3%)	0.041
Mucinous differentiation	25 (35%)	5 (17%)	0.082
Medullary differentiation	1 (1%)	0 (0%)	0.524
Tumor budding	29 (40%)	12 (41%)	0.919

N stage, M stage, pathological stage, perineural invasion, and lymphovascular invasion were similar between the two groups. T tumor stage was higher among the MSI-H group than in the MSI-L group (p = 0.014) (Table 2). MSI-H tumors were more frequently located in the right colon and transverse colon (p < 0.001).

**Table 2:** pT, pN, and pM stages and clinicopathological features in both groups. pT: primary tumor; pN: regional lymph nodes; pM: distant metastasis; MSI: microsatellite instability

Clinicopathological features	MSI-Low 72 (71%), n (%)	MSI-High 29 (29%), n (%)	P-value
Tumor localization			<0.001
Right colon	7 (10%)	15 (52%)	
Transverse colon	1 (1%)	2 (7%)	
Left colon	64 (89%)	12 (41%)	
pT			0.014
T1	2 (3%)	0	
T2	10 (14%)	12 (41%)	
T3	55 (76%)	14 (88%)	
T4	5 (7%)	3 (11%)	
pN			0.617
N0	39 (54%)	17 (59%)	
N1	23 (32%)	10 (34%)	
N2	10 (14%)	2 (7%)	
pM			0.199
M0	60 (83%)	27 (93%)	
M1	12 (17%)	2 (17%)	
Pathological stage			0.057
Stage I	12 (17%)	4 (14%)	
Stage II	24 (34%)	9 (31%)	
Stage III	24 (34%)	13 (45%)	
Stage IV	12 (17%)	3 (10%)	
Lymphovascular invasion	18 (25%)	8 (27%)	0.788
Perineural invasion	13 (18%)	4 (14%)	0.604

**Figure 2:**
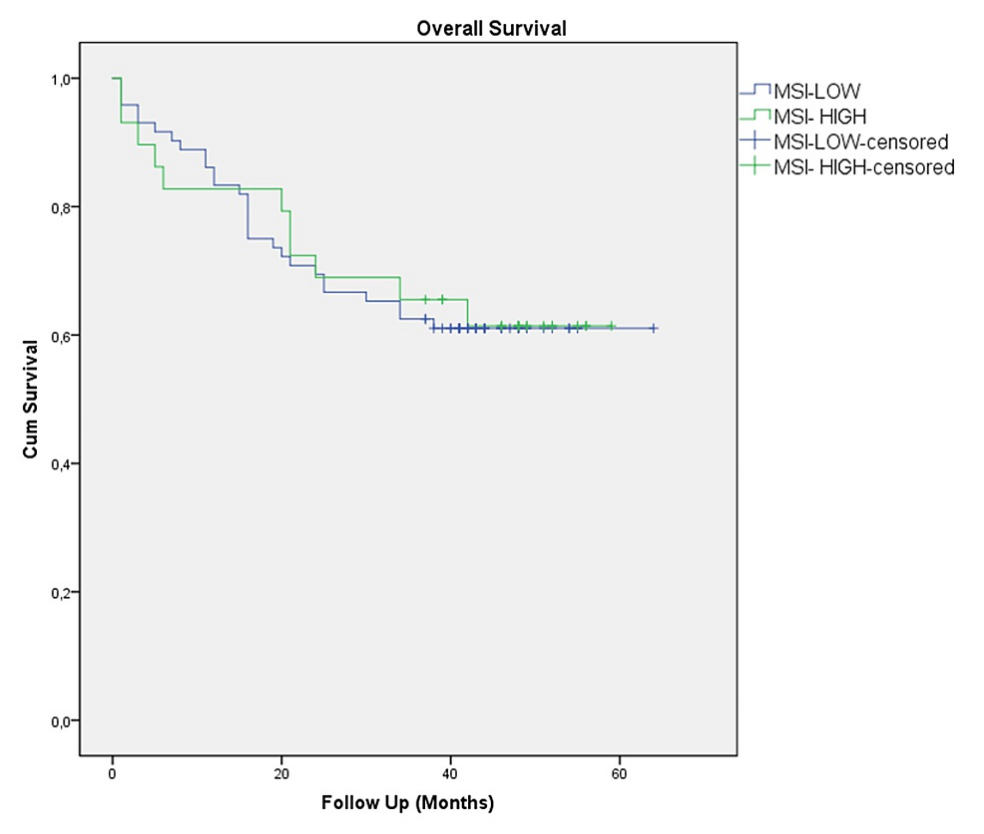
Overall survival in MSI-low and MSI-high groups (log-rank: 0.280). MSI: microsatellite instability

The mean follow-up period was 42 months (1-64 months). In the univariate and multivariate analyses, age, N stage, and M stage were independent prognostic factors for colon cancer (p < 0.001; p = 0.005; p < 0.001, respectively) (Table 3). Moreover, there was no significant effect of MSI status on colon cancer prognosis (Figure 2).

**Table 3:** Univariate and multivariate overall survival analysis for colon cancer (Cox regression). pT: primary tumor; pN: regional lymph nodes; pM: distant metastasis; MSI: microsatellite instability; HR: hazard ratio; CI: confidence interval

Clinicopathological features	HR	95% CI	P-value	HR	95% CI	P-value
Gender	1.669	0.785-3.550	0.183	-	-	-
Age	1.066	1.035-1.098	<0.001	1.184	1.046-1.120	<0.001
Histological grade	1.463	0.942-1.818	0.836	-	-	-
Border	0.306	0.094-1.016	0.053	-	-	-
Tumor localization	0.774	0.552-0.948	0.692	-	-	-
Dirty necrosis	1.381	0.649-2.936	0.402	-	-	-
Tumor-infiltrating lymphocytes	0.704	0.428-1.160	0.168	-	-	-
Crohn’s-like lymphocytic reaction	1.477	0.522-4.180	0.462	-	-	-
Mucinous differentiation	0.578	0.296-1.131	0.109	-	-	-
Tumor budding	0.921	0.477-1.778	0.806	-	-	-
pT	2.935	1.037-8.306	0.042	1.184	0.347-4.037	0.787
(T1-T2 vs. T3-T4)						
pN	3.254	1.619-6.541	0.001	3.531	1.454-8.572	0.005
(N0 vs. N+)						
pM	6.455	3.207-12.993	<0.001	5.806	2.652-12.715	<0.001
Tumor diameter	0.997	0.875-1.137	0.966	-	-	-
Lymphovascular invasion	0.463	0.237-0.906	0.025	1.026	0.464-2.266	0.95
Perineural invasion	0.836	0384-1.819	0.652	-	-	-
MSI Low/High	0.963	0.479-1.934	0.915	-	-	-

## Discussion

Understanding the pathways in the developmental biology of the tumor is essential in the patient follow-up process. Patient-based approaches are increasing due to biological differences between patient populations. Collecting data on this subject from more populations is meaningful.

CRC is the most prevalent malignancy following lung, prostate, and stomach cancers in men and breast and cervical cancer in women. It is the third leading cause of cancer-related deaths following lung and breast cancer [1]. It is clinically significant to identify CRC with MSI because it has a better prognosis, and these patients are more responsive to chemotherapy with 5-fluorouracil [4]. Molecular analysis for the definitive diagnosis of MSI-H tumors is considered time-consuming and expensive. It can be performed in centers with advanced equipment and technical support. Immunohistochemical analysis of MLH-1, PMS-2, MSH-2, and MSH-6 is faster and easier and can be used as a routine test in laboratories [12].

In the present study of 101 cases, 29 MSI-H tumors were identified by MMR immunomarkers. This incidence of 29% is higher than those reported by similar studies, including Kim et al. (13%), Lanza et al. (18.4%), Halvarsson et al. (20%), and Malik et al. (11.3%) [13-16], and similar to those reported Alexander et al. (28%) [7]. There was no significant difference between sexes by MSI in this study. In contrast, studies by Kim et al., Lanza et al., and Halvasson et al. [13-15] reported significant dominance in the female sex. In contrast, Alexander et al. and Nayak et al. [7,17] reported dominance in male participants. There was a higher incidence in the MSI-H group in tumors located in the right and transverse colon (p < 0.001). These results are consistent with previous studies by Kim et al. and Halvasson et al. [13,15], which suggested a correlation between proximal colon localization and loss of MMR expression. Kim et al., Lanza et al., and Malik et al. [13,14,16] also reported a correlation between larger tumor diameter and MSI in their respective studies. Kim et al. and Malik et al. [13,16] suggested that the sum of well and moderately differentiated tumors was significantly predominant over poorly differentiated tumors. Nevertheless, the poorly differentiated rate was significantly predominant in MSI tumors, according to the results of Lanza et al. [14]. In this study, we found no significant difference between the two groups regarding tumor diameter and histological grade. Although we considered the TIL threshold value as three lymphocytes similar to Alexander et al. [7], there was no statistically significant difference. In addition to Alexander et al. [7], who reported a high correlation between MSI and TIL, Smyrk et al. [18] suggested that the identification of five or more TILs in 10 high-magnification fields was 93% sensitive and 62% specific in determining MSI status. Previous studies reported significantly more CLR in MSI-H tumors than in MSI-L [15,16]. In this study, there was no association between CLR and the occurrence of MSI-H. However, there was an association between the MSI-L group, which contradicts the literature. We believe that the low number of cases found to have CLR in the study resulted in this finding. In the large series reported by Alexander et al. [7], TIL was a more significant parameter than CLR. In this study, there was no correlation between dirty necrosis and MMR protein expressions. Greenson et al. [6] associated the absence of dirty necrosis with MSI-H tumors in their series of 365 cases. Alexander et al. and Zumstein et al. [7,19] found no significant relationship between MMR expressions and dirty necrosis in their series. In this study, there was no correlation between medullary differentiation and MSI status. In the literature, cases without staining with MLH-1 and MSH-2 were associated with medullary morphology, as in the series of Gafa et al. [20]. The above inconsistency with the literature is because there was medullary differentiation in only one tumor in this study. There was no significant correlation between perineural and lymphovascular invasion and MSI status. In a study by Kruschewski et al. [21], MLH-1-negative cases were associated with lymphovascular invasion, whereas in the same study, this relationship was not seen in the case of MSH-2. In contrast, Wright et al. [22] reported perineural and lymphovascular invasion at a lower rate in cases with loss of MMR protein expression. There is evidence that intratumoral budding is an independent prognostic factor in colon cancer prognosis [23]. However, our study did not show a significant correlation between tumoral budding and MSI status. Future studies with larger series, including molecular assays, should investigate the relationship between tumoral budding and MSI.

This study has a few limitations. First, its retrospective design may have led to some selection biases. Second, very few patients were included in the analyses, which may not represent the entire population. The oncological treatment status of the included patients was unknown, which may mislead the survival. MSI status was not confirmed with molecular tests such as polymerase chain reaction or next-generation sequencing. Finally, the lack of power analyses may be considered another limitation.

## Conclusions

MSI-H CRCs possess distinct clinicopathological and molecular characteristics compared to MSI-L CRCs. Many factors in a routine histopathological analysis can affect prognosis. Our study presented a distribution of clinicopathological features and their relation to MSI for 101 CRC patients in the region. We found a significant relationship between proximal colon localization and higher pathological T stage with MSI-H. Age and stage N and M were independent prognostic factors for colon cancer prognosis, but MSI status had no significant effect on survival time. Identification of MSI in CRCs may help personalize therapy planning. As these characteristics may vary from population to population, further investigations are needed.
